# Optimizing drip fertigation at different periods to improve yield, volatile compounds and cup quality of Arabica coffee

**DOI:** 10.3389/fpls.2023.1148616

**Published:** 2023-06-02

**Authors:** Rongmei Li, Jinhuan Cheng, Xiaogang Liu, Zhihui Wang, Huiyong Li, Jinjin Guo, Haidong Wang, Ningbo Cui, Lu Zhao

**Affiliations:** ^1^ Faculty of Modern Agricultural Engineering, Kunming University of Science and Technology, Kunming, China; ^2^ Tropical and Subtropical Economic Crops Institute, Yunnan Academy of Agricultural Sciences, Baoshan, China; ^3^ State Key Laboratory of Hydraulics and Mountain River Engineering and College of Water Resource and Hydropower, Sichuan University, Chengdu, Sichuan, China

**Keywords:** Arabica coffee, optimized fertilization, bean nutrients, volatile compounds, cup quality

## Abstract

How to improve and regulate coffee bean yield and quality through split fertilization in the whole life cycle of coffee is still unclear and deserves further study. A field experiment of 5-year-old Arabica coffee trees was conducted for 2 consecutive years from 2020 to 2022. The fertilizer (750 kg ha^-1^ year^-1^, N-P_2_O_5_-K_2_O:20%-20%-20%) was split in three times at early flowering (FL), the berry expansion (BE), and the berry ripening (BR). Taking equal fertilization throughout the growth cycle (FL_250_BE_250_BR_250_) as the control check, variable fertilizations including FL_150_BE_250_BR_350_, FL_150_BE_350_BR_250_, FL_250_BE_150_BR_350_, FL_250_BE_350_BR_150_, FL_350_BE_150_BR_250_, and FL_350_BE_250_BR_150_. Leaf net photosynthetic rate (*A*_net_), stomatal conductance (*g*_s_), transpiration rate (*T*_r_), leaf water use efficiency (LWUE), carboxylation efficiency (CE), partial factor productivity of fertilizer (PFP), bean yield, crop water use efficiency (WUE), bean nutrients, volatile compounds and cup quality, and the correlation of nutrients with volatile compounds and cup quality was evaluated. FL_350_BE_250_BR_150_ had the maximum *A*_net_ and *g*_s_, followed by FL_250_BE_350_BR_150_. The highest dry bean yield and WUE were obtained from FL_250_BE_350_BR_150_, which increased by 8.86% and 8.47% compared with FL_250_BE_250_BR_250_ in two-year average. The ash, total sugar, fat, protein, caffeine and chlorogenic acid in FL_250_BE_350_BR_150_ were 6.47%, 9.48%, 3.60%, 14.02%, 4.85% and 15.42% higher than FL_250_BE_250_BR_250_. Cluster analysis indicated FL_150_BE_350_BR_250_, FL_250_BE_350_BR_150_, FL_350_BE_150_BR_250_ and FL_350_BE_250_BR_150_ under medium roasted degree increased pyrazines, esters, ketones and furans, FL_150_BE_350_BR_250_ and FL_250_BE_350_BR_150_ under dark roasted degree increased ketones and furans. The aroma, flavor, acidity and overall score of medium roasted coffee were higher than dark roasted coffee, while the body score of dark roasted coffee was higher than medium roasted coffee. The nutrient contents were correlated with the volatile compounds and cup quality. TOPSIS indicated that FL_250_BE_350_BR_150_ was the optimal fertilization mode in the xerothermic regions. The obtained optimum fertilization mode can provide a scientific basis for coffee fertilization optimization and management.

## 1 Introduction

The xerothermic regions of southwest China have sufficient light and heat, and a small annual temperature difference, which are very suitable for coffee cultivation. Both Arabica’s planting area and yield account for more than 98% of the coffee planting area and yield owned by China (Zhang et al., 2020). However, the poor soil and the weak ability of soil to retain water and fertilizer limit the bean quality and yield. Moreover, local people adopt one-time fertilizer application in the rainy season, resulting in soil nutrient loss, low fertilizer use efficiency, low coffee yield and quality, and many environmental pollution problems (Dinesh et al., 2010; Bruno et al., 2011; Byrareddy et al., 2019).

Soil nutrient content determines crop growth and nutrient absorption and utilization by roots, which directly affects the yield and quality of crops (Dinesh et al., 2010; Fang et al., 2018; Cai et al., 2019; Ye et al., 2020). Nitrogen is an essential nutrient in coffee and the basis for plant growth and development. The yield loss is as high as 60% when nitrogen is not applied during the coffee reproductive stage (Salamanca-Jimenez et al., 2017). Studies found that appropriate nitrogen application is beneficial to coffee root growth and nutrient uptake, promotes chlorophyll synthesis, enhances net photosynthetic rate (*A*_net_) and stomatal conductance (*g*_s_), improves nitrogen use efficiency and increases yield of coffee beans (Bruno et al., 2011; Salamanca-Jimenez et al., 2017; Zhang et al., 2017). Potassium fertilizer is indispensable in the growth and maturation of coffee fruit as it can promote cell differentiation and protein and carbohydrate synthesis (Silva et al., 2003; Ali et al., 2021). Although, the effect of phosphate fertilizer on coffee growth, photosynthesis and yield is not as vital as nitrogen and potassium fertilizer, it plays an important role in promoting coffee growth and mineral nutrient absorption (Cai et al., 2007). Zhang et al. (2017) showed that the plant height, stem diameter growth rate, *A*_net_, *g*_s_ and intrinsic water use efficiency of coffee saplings under medium and high fertilizer treatment were higher than those under low fertilizer treatment, and it was found that the growth indexes of coffee were superexcellent when the application of nitrogen, phosphorus and potassium was 1:0.8:0.5. Liu et al. (2016) found that fertilization could significantly increase the contents of caffeine, protein and chlorogenic acid in coffee beans. Król et al. (2020) found that coffee beans obtained by using organic fertilizer contain more bioactive substances (total phenols, phenolic acids, flavonoids, etc.), while the excessive use of chemical fertilizers resulted in more caffeine in coffee beans. The above studies indicated that fertilization could meet the nutrient requirements of coffee vegetative and reproductive growth, promote photosynthetic physiology, and improve yield and quality of beans (Bruno et al., 2011; Liu et al., 2016; Król et al., 2020). Previous studies have shown that fertilization can increase the abundance of volatile compounds in fruit, such as tomato, strawberry and apple (Raffo et al., 2014; Cvelbar Weber et al., 2021; Pasković et al., 2021). The effects of cultivars, planting area, altitude, climate, processing method and roasting degree, etc. on the volatile flavor of coffee have been reported (Sunarharum et al., 2014; Dong et al., 2019; Tassew et al., 2021; Zakidou et al., 2021), but little is known about the effect of fertilization on volatile compounds.

One-time fertilization, excessive fertilization or unsystematic fertilization tend to reduce fertilizer use efficiency, leading to low productivity (Yan et al., 2021). Bruno et al. (2015) found that the most effective nitrogen use efficiency in coffee was 200 kg ha^-1^ year^-1^ by studying the nitrogen use efficiency of different fertilizer application amount. Leal et al. (2010) found that at least 30% of nitrogen in urea would be lost by volatilization when 360 kg ha^-1^ urea was applied in coffee. Drip fertigation can synchronously and evenly transport water and fertilizer to crop roots, reduce water evaporation and nutrient leakage, and thus achieve efficient utilization of water and fertilizer (Guo et al., 2022; Sun et al., 2022). Drip fertigation can promote crop growth and improve leaf photosynthetic capacity by changing soil microenvironment, thus increasing crop yield, quality and water, and fertilizer use efficiency (Sakai et al., 2015; Vinecky et al., 2017; Geisseler et al., 2020; Rakocevic et al., 2023). Split fertilization under drip fertigation significantly affects the availability of soil nutrients in the root zone of crops, promotes the absorption of nutrients and the effective allocation of photosynthetic assimilates, which is conducive to high yields of crops (Bartholo et al., 2003; Parvizi and Sepaskhah, 2015; Sun et al., 2022). Previous studies have shown that split fertilization can increase crop nutrient absorption efficiency, improve photosynthesis, and coordinate organic matter distribution in various organs of crops, which is conducive to starch synthesis and accumulation, increase vitamin C and soluble sugar content, and further facilitate crop yield and fruit quality (Wang et al., 2008; Bruno et al., 2011; Corrêa et al., 2018; Byrareddy et al., 2019). With the same amount of fertilizer applied during the mango growth period, increasing the fertilizer application rate at fruit expansion could improve mango yield and partial fertilizer productivity, and increasing the fertilizer application at fruit ripening could increase the content of vitamin C, carotenoids and soluble solids in mango (Sun et al., 2022). The fertilizer requirement of coffee was found to be closely related to the physiological stages (Bartholo et al., 2003). Bruno et al. (2011) found that the leaves have the greatest demand for nitrogen fertilizer during the fruit expansion of coffee. Sun et al. (2018) indicated that the application of nitrogen fertilizer at the fruit expansion period was beneficial to increasing the dry matter accumulation of *Coffea canephora*, while excessive application of nitrogen fertilizer would reduce fertilizer use efficiency. It is not clear about the amount of fertilizer required for adult coffee trees in each growth period, but it is well known that fertilization during coffee berry expansion is most conducive to increasing fruit yield. At present, studies about coffee fertilization have focused on the effects of different nitrogen application rates or fertilizer ratios on growth, yield and quality (Dinesh et al., 2010; Bruno et al., 2015; Sakai et al., 2015; Sun et al., 2018; Byrareddy et al., 2019). However, there are few reports about the effects of split fertilization on photosynthetic physiology, yield, quality and volatiles of coffee.

How to improve and regulate coffee bean yield and quality through split fertilization is still unclear and deserves further study. We hypothesized that different fertilizer application at early flowering, berry expansion and berry ripening would affect coffee leaf gas exchange, bean yield and quality. In this experiment, the effects of drip fertigation on leaf gas exchange, bean nutrients, volatile components and cup quality of Arabica coffee were studied. The aim was to find the best combination of drip irrigation and split fertilization mode, and provide practical basis for nutrient management, high-quality and high-yield of Arabica coffee in the xerothermic regions.

## 2 Materials and methods

### 2.1 Experimental site

**Figure 1:**
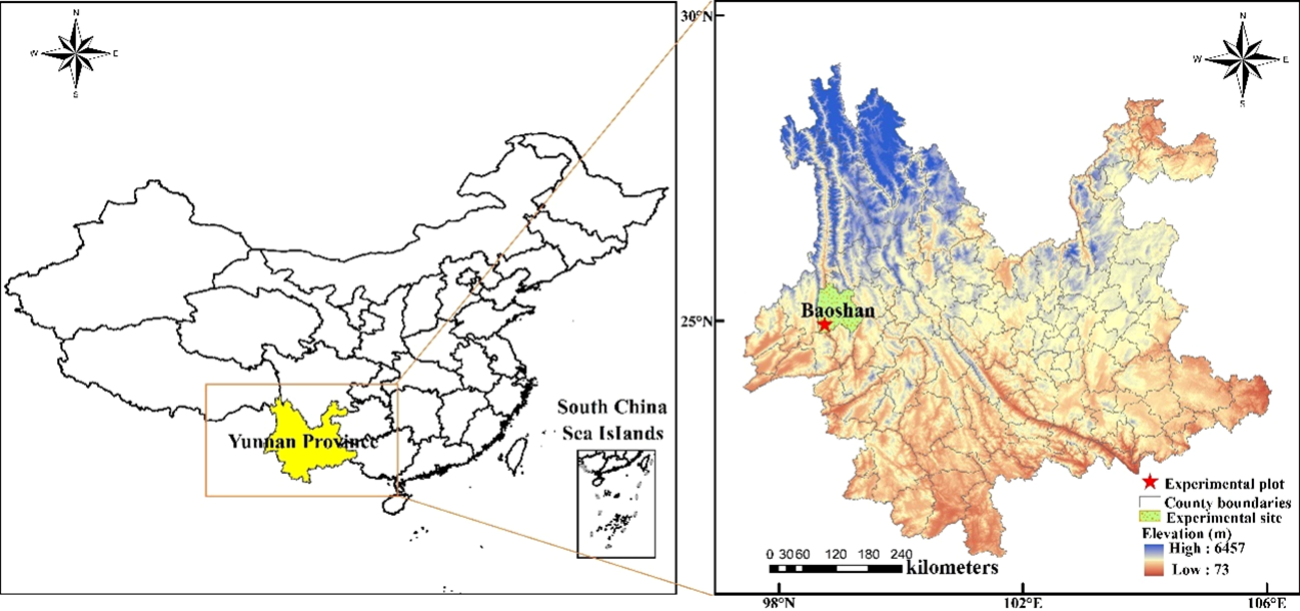
Geographical location of the experimental site.

**Figure 2:**
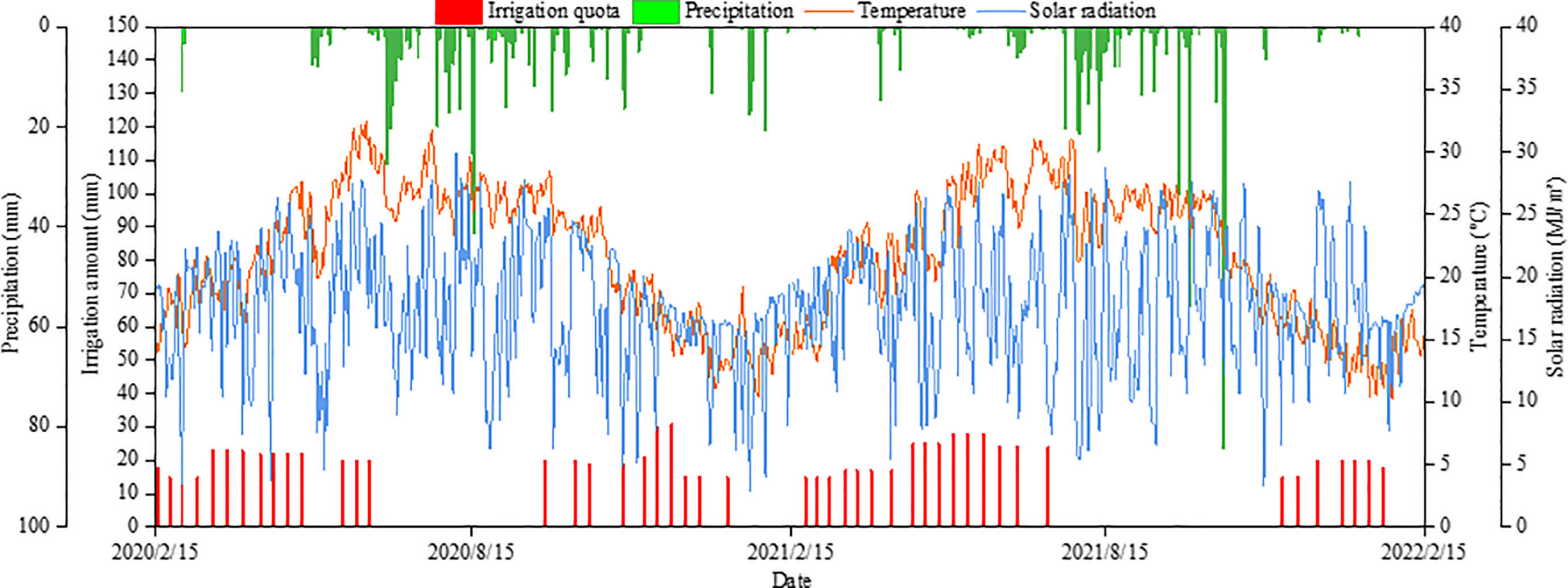
The solar radiation, daily temperature, precipitation and irrigation quota during the experiment.

The field experiment was conducted from February 2020 to February 2022 in Lujiangba, Baoshan, Yunnan Province, Southwest China (25°4′N, 99°11′E; 799.50 m above sea level) (**Figure 1**). The region has a typical hot-dry valley climate, with average annual precipitation of 755.40 mm (80% concentrated in June to October), average annual evaporation of 2101.90 mm, average annual temperature of 21.3°C, absolute maximum and minimum temperatures of 40.4°C and 0.2°C, average annual sunshine hours of 2328 h, and a relative humidity of 71%. The precipitation was 583.3 mm in 2020-2021 and 575.8 mm in 2021-2022, and the average daily minimum and maximum temperatures were 10.22°C and 32.49°C (**Figure 2**). The soil in the experimental field was red-brown sandy loam, with organic matter of 17.0 g kg^-1^, total nitrogen of 1.1 g kg^-1^, total phosphorus of 1.15 g kg^-1^, available nitrogen of 90.6 mg kg^-1^, available phosphorus of 12.7 mg kg^-1^, and available potassium of 126.7 mg kg^-1^.

The 5-year-old coffee trees (Catimor CIFC7963) with uniform growth potential (1.65-1.85 m of height) were used as the experimental material. The row spacing was 1.5 m × 2.0 m.

### 2.2 Experiment design

The total fertilization amount was determined to be 750 kg ha^-1^ year^-1^ referring to the local fertilization practices and split in three times at early flowering (FL: March), the berry expansion (BE: July), and the berry ripening (BR: October). There were 7 fertilization modes: equal fertilization throughout the growth cycle (FL_250_BE_250_BR_250_) and 6 variable fertilizations (FL_150_BE_250_BR_350_, FL_150_BE_350_BR_250_, FL_250_BE_150_BR_350_, FL_250_BE_350_BR_150_, FL_350_BE_150_BR_250_, FL_350_BE_250_BR_150_). Each treatment had 3 replicates, in which consisted of 5 trees, each replicate was an experimental plot with an area of 15 m^2^ (2 m × 7.5 m), and 21 plots in total. Macronutrient water-soluble fertilizer (N-P_2_O_5_-K_2_O:20%-20%-20%) (Xuelvfeng, Saigute Biotechnology Co., Ltd., Wuhan, China) was adopted and applied on 5^th^ March, 20^th^ July and 25^th^ October in 2020, 8^th^ March, 25^th^ July, and 29^th^ October in 2021. Drip fertigation was carried out with a differential pressure fertilization tank.

The irrigation quota was determined based on monthly water consumption intensity data in local Arabica coffee (Chen et al., 1995) and effective precipitation in the region. The equation 1:


Eq. 1 $$I_{i}=(ET_{ci}\times n)-P_{i}$$

where *I_i_* is irrigation quota in the ith period (mm), *ET_ci_* is average water consumption intensity in the *i*th period (mm d^−1^), *n* is the period (d), and *P_i_* is effective precipitation in the *i*th period (mm).

The field irrigation system was equipped with a water pumping station, a filter device, a main pipeline, branch pipelines, capillary pipelines, and emitters. The branch control method was adopted to design drip irrigation pipelines, and the irrigation amount was controlled by the water meter. The flow rate of emitters was 2.5 L h^-1^ with a working pressure of 0.3 MPa. One circular capillary was set 0.3 m away from the trunk, and each circular capillary installed 2 emitters. All treatments were fully irrigated every 7 days during the experiment, with a total irrigation quota of 957.0 mm. The precipitation and irrigation quota are shown in **Figure 2**. Manual tillage and weeding were carried out monthly, pest controlled in early May, there was no shaping and pruning of coffee trees. Other field management was consistent with local practice.

### 2.3 Measurements and calculations

#### 2.3.1 Leaf gas exchange measurements

The *A*_net_, transpiration rate (*T*_r_), *g*_s_ of healthy functional leaves of the 3^rd^ to 5^th^ pairs in the superior plant layer counted from the tip was measured using a portable photosynthesis system (LI-6400XT, Lincoln, Nebraska, USA) at 9:00-11:00 am (under natural irradiance) on 22^nd^ May, 5^th^ October, 21^st^ December in 2020 and 26^th^ May, 5^th^ October, 23^rd^ December in 2021.

Leaf water use efficiency (LWUE) was calculated (Li et al., 2021) as equation 2:


Eq. 2 $$LWUE=\frac{A_{\text{net}}}{T_{\text{r}}}$$

Where LWUE is leaf water use efficiency (μmol mmol^-1^), *A*_net_ is leaf net photosynthetic rate (µmol m^-2^ s^-1^), *T*_r_ is transpiration rate (mmol m^-2^ s^-1^).

Carboxylation efficiency (CE) was calculated (Liu et al., 2019) as equation 3:


Eq. 3 $$CE=\frac{A_{\text{net}}}{C_{\text{i}}}\times 1000$$

Where CE is carboxylation efficiency (mmol m^-2^ s^-1^), *C*_i_ is intercellular CO_2_ concentration (µmol mol^-1^).

#### 2.3.2 Dry bean yield

Fresh bright-red mature beans were harvested by hand in batches from December to February of the next year. The fresh beans were decorticated with a coffee decorticator and soaked in water. Then the beans were washed, kneaded, shelled and degummed. The bean yield (kg ha^-1^) was determined after dried in natural sunlight until the water content was about 11%-12%. Appropriate amount of coffee beans harvested in 2021-2022 were selected to determine the nutrients of green coffee beans, volatile compounds and cup quality of roasted beans.

#### 2.3.3 Crop water use efficiency and partial factor productivity of fertilizer

Crop water use efficiency (WUE) was calculated (Liu et al., 2016) as equation 4:


Eq. 4 $$WUE=\frac{Y}{ET}$$

where WUE is crop water use efficiency (kg m^−3^), *Y* is coffee dry bean yield (kg ha^−1^), and *ET* is total water consumption over the growing period (mm). *ET* was calculated as equation 5:


Eq. 5 $$ET=I+P_{r}-\Delta W$$

where *I* is total irrigation quota (mm), *P_r_* is effective precipitation (mm), and *ΔW* is variation of soil moisture during the experimental period (mm).

Partial factor productivity of fertilizer (PFP) was calculated (Cui et al., 2020) as equation 6:


Eq. 6 $$PFP=\frac{Y}{F}$$

Where PFP is partial factor productivity of fertilizer (kg ^−1^), *Y* is total dry bean yield of coffee (kg ha^−1^), *F* is the total input of fertilizer (kg ha^−1^).

#### 2.3.4 Nutritional components

The appropriate amount of shelled coffee beans was crushed with a grinder (WK-1000A, Shandong Jingcheng Medicine Equipment Manufacturing Co., Ltd., Shandong, China) to determine their nutrients. Ash content was determined by the muffle furnace method. About 5.00 g of coffee sample was placed into a muffle furnace (M10L1200, Sigma (Shanghai) High Temperature Electric Furnace Co., Ltd., Shanghai, China) at 600 ± 25°C to completely carbonizing till smokeless, cooling and weighing. Protein was determined by Kjeldahl method. Weighing fully mixed grinded coffee samples 0.20-0.30 g into the digestive tube, adding a mixed catalyst of copper sulfate and potassium sulfate and 20 mL of sulfuric acid in a fume hood for digestion and carbonization. Automatic liquid addition, distillation, titration and recording with automatic Kjeldahl nitrogen analyzer (K1100, Haineng Future Technology Group Co. Ltd, Shandong, China). Fat was determined by Soxhlet extraction method. Weighing an evenly mixed coffee sample of 5.00 g in a Soxhlet extractor (BSXT06-150, Beijing Haifuda Technology Co., Ltd., Beijing, China), extracting 6-8 h for 70°C water bath, siphoning every 3-5 min. Total sugar was determined by anthrone colorimetric method. 0.60 mL of the prepared extract was added with 2.40 mL of anthrone reagent. Standard curves were measured with 0.10 mg mL^-1^ glucose standard solution at 620 nm wavelength by spectrophotometer (UV-1600, Shanghai Meifuda Instrument Co., Ltd., Shanghai, China). Caffeine and chlorogenic acid were determined by ultraviolet spectrophotometry (Khochapong et al., 2021). The prepared coffee sample extract was mixed with dichloromethane at a ratio of 1:1. After 10 min of shaking, caffeine and chlorogenic acid were separated using a separatory funnel. The absorbance of chlorogenic acid (dissolved in distilled water) was determined by spectrophotometer at a wavelength of 324 nm with distilled water as blank. The caffeine extract (dissolved in dichloromethane) was determined by spectrophotometry at a wavelength of 274 nm with dichloromethane as a blank.

#### 2.3.5 Coffee bean roasting and volatile compounds

120 g of green coffee beans were poured into a coffee baking machine (TN-100-1G, VINA NHATRANG, Vietnam) with the pot temperature of 180°C, the firepower of 1 Kw, and the damper of 0.5. Adjusting the firepower to 1.5 Kw when the bean temperature reached 170°C, then reduced the firepower to 0.8 Kw after the first explosion began at 193°C, held for 2 min, and took out when the temperature reached 208°C to obtain coffee samples with medium roasting degrees. After the first explosion under the same conditions, held for 3 min, and took out when the temperature reached 225°C to obtain coffee samples with dark roasting degrees. Then the cooling device was opened 1 min before flameout, and the coffee samples were quickly cooled to room temperature. The roasted coffee beans were crushed and passed through 40 mesh sieves, and then the volatile compounds were measured.

Headspace-gas chromatography-mass spectrometry (HS-GC-MS) described by Dong et al. (2019) was used for the analysis of volatile compounds. The solid-phase microextraction probe (SPME) (50/30 μm CAR/PDMS/DVB, Supelco, USA) was inserted into the injection port of GCMS-QP2010 gas chromatography-mass spectrometer (Shimadu, Japan) and aged for 1 h at 300°C. Up to 1.5 g of roasted ground coffee was placed in a 10 mL vial, equilibrated with a thermostatic heater at 60°C for 20 min, extracted for 30 min using the SPME fiber in the headspace, and then injected into the 250°C injection port for desorption for 3 min. Volatile compounds were separated using a polar DB-WAX capillary column (30 m × 0.25 mm × 0.25 μm film thickness). The oven temperature was maintained at 40°C for 2 min, raised at 1.5°C/min to 130°C, and then increased at 4°C/min up to 200°C and held for 5 min. The detector temperature was 230°C, and the injection port temperature was 250°C. The carrier gas was helium without split injection, with a flow rate of 1.0 mL/min for 3 min. Mass spectrometry analysis operated in the electron impact ionization mode (70 eV), with a scan range of 35-350 amu and ion source temperature of 230°C.

The compounds were tentatively identified by comparing the mass spectra based on a similarity search more than 80% of NIST05 library as well as related literature. The peak area normalization method was used for quantitative analysis (Dong et al., 2018).

#### 2.3.6 Cup quality

The roasted coffee beans were naturally cooled and ground into fine powder with an average particle size of about 0.60-0.70 mm. Then 10.00 g roasted ground coffee was added into the cup and about 180 mL purified water at 90°C was poured on the cup for 4 min (Worku et al., 2018). Referring to the method of Hu et al. (2020), coffee cup quality was determined by five professional evaluators. The sensory scoring indexes included aroma, flavor, aftertaste, acidity, body, balance, overall, cleanliness, uniformity, and sweetness. The sensory indexes were evaluated in the range of 0 to 10 with the increment of 0.25, and the sum of 10 sensory indexes was the total score of an individual sample.

### 2.4 TOPSIS comprehensive scoring method

Technique for order preference by similarity to ideal solution (TOPSIS) is a commonly used comprehensive evaluation method. It can make full use of the information of the original data, and the results can accurately reflect the gap between the evaluation schemes. According to the improved TOPSIS, the comprehensive benefit evaluation model of coffee physiological indexes, nutritional quality indexes and coffee cups was established (Liu et al., 2016; Sun et al., 2022). Steps are as follows:

(1) Establishing the matrix *R* of evaluation objects and evaluation indicators. There were 7 evaluation objects (m) of fertilization, and 34 evaluation indexes (n) in 2021-2022 for *A*_net_, *T*_r_, *g*_s_, LWUE, CE, bean yield, bean quality (ash, total sugar, fat, protein, caffeine and chlorogenic acid), and indexes of coffee cups with different roasting degrees (aroma, flavor, aftertaste, acidity, body, balance, overall, cleanliness, uniformity, sweetness and total score), using the equation 7:


Eq. 7 $$R={\left(r_{ij}\right)}_{m\times n}$$

where *r_ij_* is the *j*th evaluation index under the *i*th evaluation object, m = 7, n = 34.

(2) Indicators were normalized to build a normative decision matrix *Z*=(*z_ij_*)*_m_*_×_ *_n_*, using the equation 8:


Eq. 8 $$Z_{ij}=r_{ij}\cdot {\left(\sum_{i=1}^{m}(r_{ij}{)}^{2}\right)}^{-0.5}$$

(3) Weight determination:

(a) The judgment matrix was constructed using the equation 9:


Eq. 9 $$B={\left(b_{xp}\right)}_{n\times m}={\left(z_{ji}\right)}_{n\times m}$$

where *b_xp_* is the *p*th evaluation object under the *x*th evaluation index of the matrix.

(b) Each column of the judgment matrix was normalized using the equation 10:


Eq. 10 $$b_{xp}^{'}=b_{xp}\cdot {\left(\sum_{k=1}^{n}b_{xp}\right)}^{-1}$$

where *k* represents the *k*th evaluation index.

(c) The matrix was summed by rows using the equation 11:


Eq. 11 $${\bar{w}}_{x}=\sum_{p=1}^{m}b_{xp}^{'}$$

(d) The vector $\bar{W}={\left[\bar{w_{1}},\bar{w_{2}},\cdot \cdot \cdot ,\bar{w_{x}}\right]}^{\text{T}}$ was normalized using the equation 12:


Eq. 12 $$w_{x}={\bar{w}}_{xp}\cdot {\left(\sum_{p=1}^{n}{\bar{w}}_{p}\right)}^{-1}$$

(4) The weighting matrix *Z*′ was constituted using the equation 13:


Eq. 13 $$z_{ij}^{'}=w_{x}\cdot z_{ij}$$

(5) The positive ideal solution ( $z_{i}^{+}$ ) and the negative ideal solution ( $z_{i}^{-}$ ) were obtained using the equation 14 and 15:


Eq. 14 $$z_{i}^{+}=\underset{1\leq i\leq m}{\max}z_{ij}^{'}$$

Eq. 15 $$z_{i}^{-}=\underset{1\leq i\leq m}{\min}z_{ij}^{'}$$

(6) The Euclidean distances of $D_{i}^{+}$ and $D_{i}^{-}$ between each object and $z_{i}^{+}$ $z_{i}^{-}$ were calculated using the equation 16 and 17:


Eq. 16 $$D_{i}^{+}={\left(\sum_{i=1}^{m}(z_{ij}^{'}-z_{j}^{+}{)}^{2}\right)}^{0.5}$$

Eq. 17 $$D_{i}^{-}={\left(\sum_{i=1}^{m}(z_{ij}^{'}-z_{j}^{-}{)}^{2}\right)}^{0.5}$$

(7) The relative proximity coefficients were calculated and sorted using the equation 18. The closer *R_c_* is to 1, the better the evaluation object is.


Eq. 18 $$R_{c}=\frac{D_{i}^{-}}{D_{i}^{+}+D_{i}^{-}},(0\leq R_{c}\leq 1)$$

### 2.5 Statistical analysis

To realize data visualization, hierarchical clustering was used to investigate the variation of flavor substances among different fertilizer treatments. hierarchical clustering creates a hierarchical nested clustering tree by calculating the similarity between data points of different categories. The merging algorithm of hierarchical clustering combines the two most similar data points of all data by calculating the similarity between two categories of data points, and repeats this process. Each class of compounds was normalized in the study. Moreover, square Euclidean distance was used to perform similarity measure, and the data points with the smallest distance value were combined each other. The clustering algorithm was ward’s linkage method that minimizes the sum of squares of all clusters.

**Figure 3:**
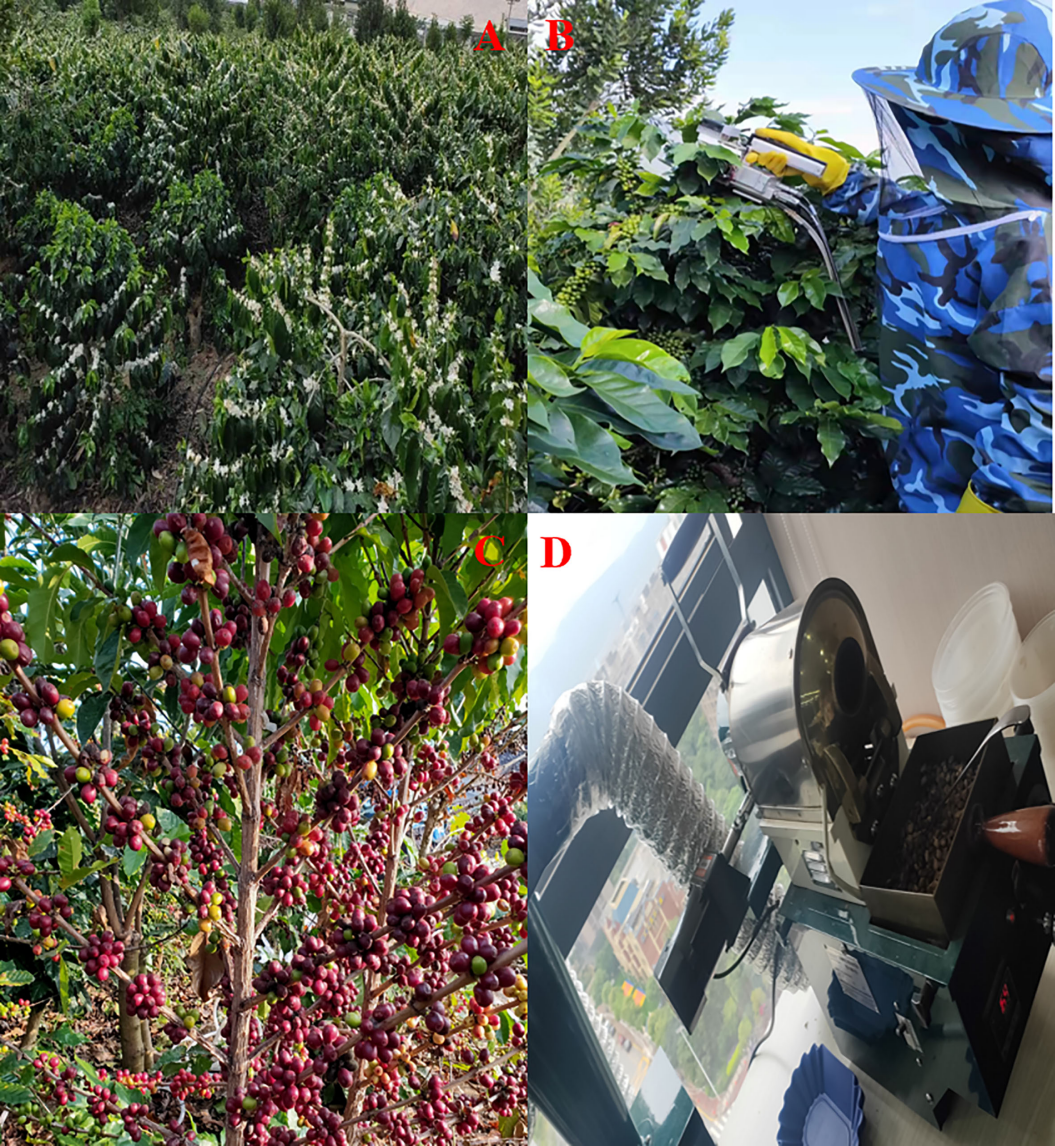
Pictures of experiment. **(A)** experimental field, **(B)** Leaf gas exchange measurements, **(C)** Berry maturity, **(D)** Coffee bean roasting.

Data collecting and collating were conducted by Excel 2010. TOPSIS was executed by Excel 2010. Graphics drawing and cluster analysis was performed by Origin 2018 software. One-way analysis of variance (ANOVA) (*P*<0.05) and Pearson correlation analysis were performed using SPSS 23.0 software. Part of experimental pictures are shown in **Figure 3**.

## 3 Results

### 3.1 Leaf gas exchange

**Figure 4:**
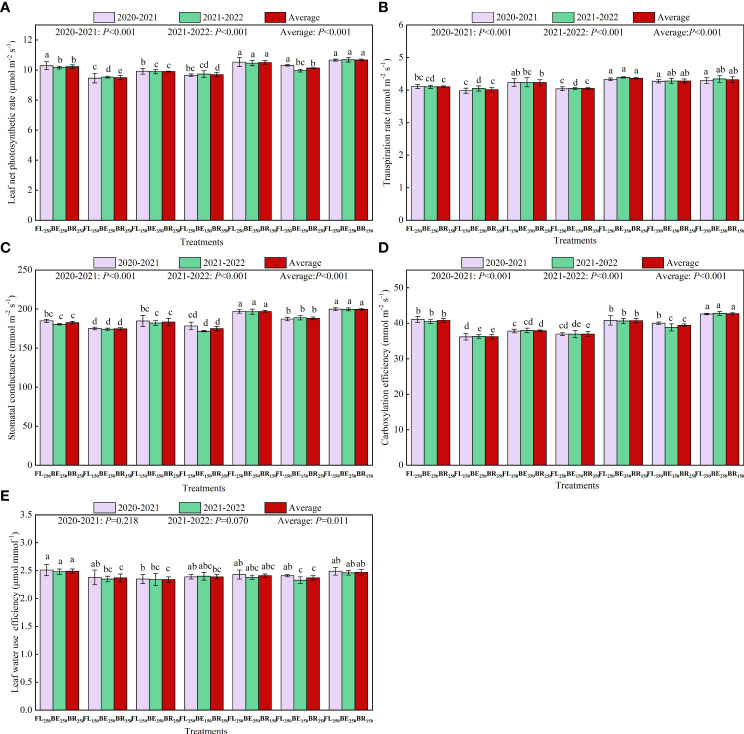
Effects of split fertilization on leaf net photosynthetic rate **(A)**, transpiration rate **(B)**, stomatal conductance **(C)**, carboxylation efficiency **(D)** and leaf water use efficiency **(E)** of Arabica coffee. Different lowercase letters at the top of the bar indicate significant differences among treatments (*P*<0.05).

Different fertilization mode had significant effects on *A*_net_, *T*_r_, *g*_s_ and CE of Arabica coffee. Among them, FL_350_BE_250_BR_150_ had the highest *A*_net_ and *g*_s_, with a two-year average value of 10.67 µmol m^-2^ s^-1^ and 199.77 mmol m^-2^ s^-1^, respectively, while FL_150_BE_250_BR_350_ had the lowest *A*_net_ and *g*_s_ (**Figures 4A, C**). FL_250_BE_350_BR_150_ had the highest *T*_r_ (**Figure 4B**), with a two-year average value of 4.36 mmol m^-2^ s^-1^. In 2020-2021, compared with FL_250_BE_250_BR_250_, FL_150_BE_250_BR_350_ decreased *A*_net_ and *g*_s_ by 8.07% and 5.26%, FL_250_BE_150_BR_350_ decreased *A*_net_ by 6.22%, FL_250_BE_350_BR_150_ increased *T*_r_ and *g*_s_ by 5.35% and 6.53%, FL_350_BE_250_BR_150_ increased *g*_s_ by 8.12%. In 2021-2022, compared with FL_250_BE_250_BR_250_, FL_150_BE_250_BR_350_ decreased *A*_net_ by 6.30%, FL_250_BE_350_BR_150_ increased *T*_r_ and *g*_s_ by 7.07% and 8.97%, FL_350_BE_250_BR_150_ increased *A*_net_, *T*_r_ and *g*_s_ by 5.16%, 5.85% and 10.62%, respectively. For the two-year average, compared with FL_250_BE_250_BR_250_, FL_150_BE_250_BR_350_ and FL_250_BE_150_BR_350_ decreased *A*_net_ by 7.23% and 5.28%, FL_250_BE_350_BR_150_ increased *A*_net_, *T*_r_ and *g*_s_ by 7.92%, 6.34% and 7.74%, FL_350_BE_250_BR_150_ increased *A*_net_, *T*_r_ and *g*_s_ by 5.28%, 5.37%, and 9.63%. Except for FL_350_BE_250_BR_150_, other treatments decreased CE compared with FL_250_BE_250_BR_250_ (**Figure 4D**). Compared with FL_250_BE_250_BR_250_, FL_150_BE_250_BR_350_, FL_150_BE_350_BR_250_ and FL_250_BE_150_BR_350_ decreased CE by 12.02%, 8.00% and 10.07% in 2020-2021, and decreased CE by 11.15%, 7.09% and 9.41% of two-year average, respectively. The LWUE value of FL_250_BE_250_BR_250_ was the highest, with a two-year average of 2.49 μmol mmol^-1^ (**Figure 4E**).

### 3.2 Dry bean yield

**Figure 5:**
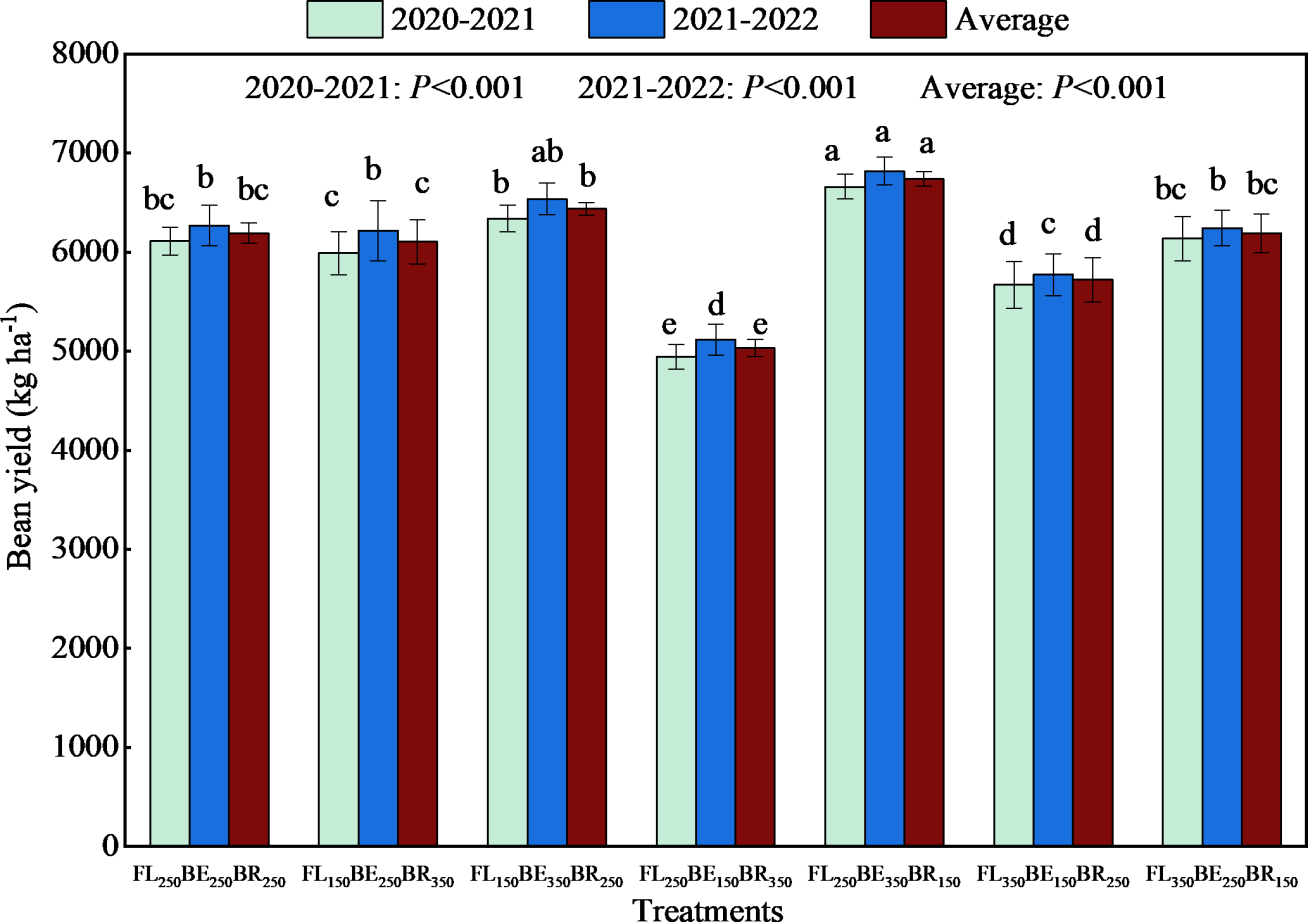
Effects of split fertilization on dry bean yield of Arabica coffee. Different lowercase letters at the top of the bar indicate significant differences among treatments (*P*<0.05).

Different fertilization mode significantly influenced the yield of Arabica coffee. In both years, the highest yield occurred in FL_250_BE_350_BR_150_ and the lowest yield occurred in FL_250_BE_150_BR_350_ (**Figure 5**). Compared with FL_250_BE_250_BR_250_, the yield of FL_250_BE_150_BR_350_ and FL_350_BE_150_BR_250_ were decreased by 19.11% and 7.24% in 2020-2021, decreased by 18.38% and 7.92% in 2021-2022, and two-year average yield decreased by 18.74% and 7.58%. However, the yield of FL_250_BE_350_BR_150_ in 2020-2021, 2021-2022 and two-years average were 8.99%, 8.74% and 8.86% higher than those in FL_250_BE_250_BR_250_, respectively. Furthermore, there was no significant difference in yield among FL_250_BE_250_BR_250_, FL_150_BE_250_BR_350_, FL_150_BE_350_BR_250_ and FL_350_BE_250_BR_150_.

### 3.3 Crop water use efficiency and partial factor productivity of fertilizer

**Figure 6:**
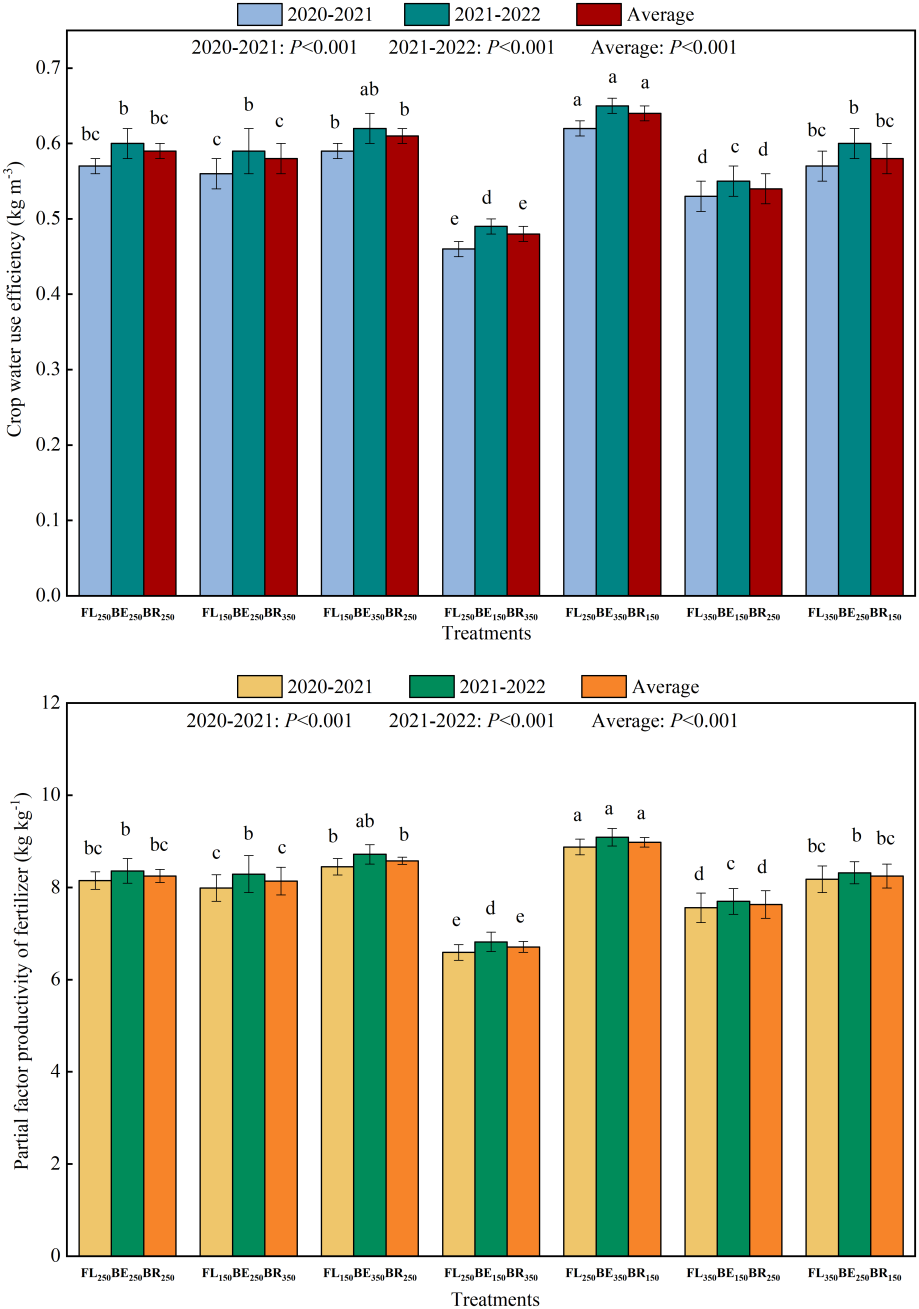
Effects of split fertilization on crop water use efficiency **(A)** and partial factor productivity of fertilizer **(B)** of Arabica coffee. Different lowercase letters at the top of the bar indicate significant difference among treatments (*P*<0.05).

Different fertilization mode had significant effect on crop water use efficiency (WUE) of Arabica coffee in two years. As could be seen from **Figure 6A**, there was no significant difference on WUE between FL_150_BE_250_BR_350_, FL_150_BE_350_BR_250_, FL_350_BE_250_BR_150_ and FL_250_BE_250_BR_250_. Compared with FL_250_BE_250_BR_250_, FL_250_BE_150_BR_350_ and FL_350_BE_150_BR_250_ decreased average WUE by 18.64% and 8.47%, while FL_250_BE_350_BR_150_ increased by 8.47%.

In terms of PFP, FL_250_BE_150_BR_350_ had the lowest PFP, and both FL_150_BE_350_BR_250_ and FL_250_BE_350_BR_150_ had higher PFP (**Figure 6B**), indicating that higher fertilization in BE was conducive to improving fertilizer utilization efficiency.

### 3.4 Nutritional components

Different fertilization had significant effects on bean nutrient content of Arabica coffee. FL_250_BE_350_BR_150_ had the highest nutrient content except of fat and caffeine, while FL_250_BE_150_BR_350_ had the lowest except of caffeine and chlorogenic acid (**Table 1**). Compared with FL_250_BE_250_BR_250_, F_I150_F_II350_F_III250_ and F_I250_F_II350_F_III150_ increased ash content by 1.98% and 6.47%, increased total sugar content by 5.23% and 9.68%, increased fat content by 5.77% and 3.60%, increased protein content by 7.57% and 14.02%, increased caffeine content by 18.45% and 4.85%, and increased chlorogenic acid content by 11.46% and 15.42%. Compared with FL_250_BE_250_BR_250_, FL_250_BE_150_BR_350_ and FL_350_BE_150_BR_250_ decreased ash content by 10.97% and 10.79%, decreased total sugar content by 9.07% and 6.19%, decreased fat content by 3.73% and 3.39%, decreased protein content by 7.05% and 6.65%, and decreased chlorogenic acid content by 12.64% and 14.96%. The caffeine content of FL_350_BE_150_BR_250_ and FL_350_BE_250_BR_150_ decreased by 7.77% and 6.80% compared with FL_250_BE_250_BR_250_. There was no significant difference on nutrient content among FL_150_BE_250_BR_350_, FL_350_BE_250_BR_150B_ and FL_250_BE_250_BR_250_ except of ash content.

**Table 1:** Effects of fertilization modes at different growth stages on bean nutrients.

Treatment	Ash /g (100g)^-1^	Total sugar /g (100g)^-1^	Fat /g (100g)^-1^	Protein /g (100g)^-1^	Caffeine /%	Chlorogenic acid /g kg^-1^
FL_250_BE_250_BR_250_	5.56±0.12b	11.47±0.10d	14.73±0.07cd	15.33±0.19c	1.03±0.02bc	23.73±1.01b
FL_150_BE_250_BR_350_	5.27±0.13c	11.28±0.21d	14.59±0.15d	15.23±0.16c	1.12±0.08b	23.23±0.69b
FL_150_BE_350_BR_250_	5.67±0.14ab	12.07±0.10b	15.58±0.22a	16.49±0.33b	1.22±0.04a	26.45±0.77a
FL_250_BE_150_BR_350_	4.95±0.20d	10.43±0.11f	14.18±0.08e	14.25±0.17d	1.05±0.04b	20.73±0.80c
FL_250_BE_350_BR_150_	5.92±0.20a	12.58±0.09a	15.26±0.23b	17.48±0.26a	1.08±0.04b	27.39±0.45a
FL_350_BE_150_BR_250_	4.96±0.10d	10.76±0.13e	14.23±0.16e	14.31±0.13d	0.95±0.04c	20.18±1.02c
FL_350_BE_250_BR_150_	5.22±0.16cd	11.77±0.10c	14.95±0.19c	15.17±0.22c	0.96±0.03c	23.65±0.64b
Significance test (*P* value)	<0.001	<0.001	<0.001	<0.001	<0.001	<0.001

Values are means ± standard deviations (n=3), different lowercase letters in the same column indicate significant difference at *P*<0.05 level.

### 3.5 Volatile compounds

#### 3.5.1 Volatile compounds of roasted coffee beans at various fertilization modes

The volatile compounds and relative contents of medium (M) and dark (D) roasted coffee under different fertilization modes are shown in **Supp. S1**. The results showed that 81 volatile compounds were identified in roasted ground coffee, including 15 furans, 14 ketones, 2 aldehydes, 8 esters, 2 alcohols, 6 phenols, 2 acids, 12 pyrazines, 6 pyridines, 7 pyrroles and 7 others. Medium roasted beans detected 63-73 volatiles and dark roasted beans detected 69-75 volatiles. There were 54 same volatile compounds accounting for 81.00-91.00% in coffee beans under different fertilization treatments, which constituted the main flavor of coffee (**Supp. S1**).

**Figure 7:**
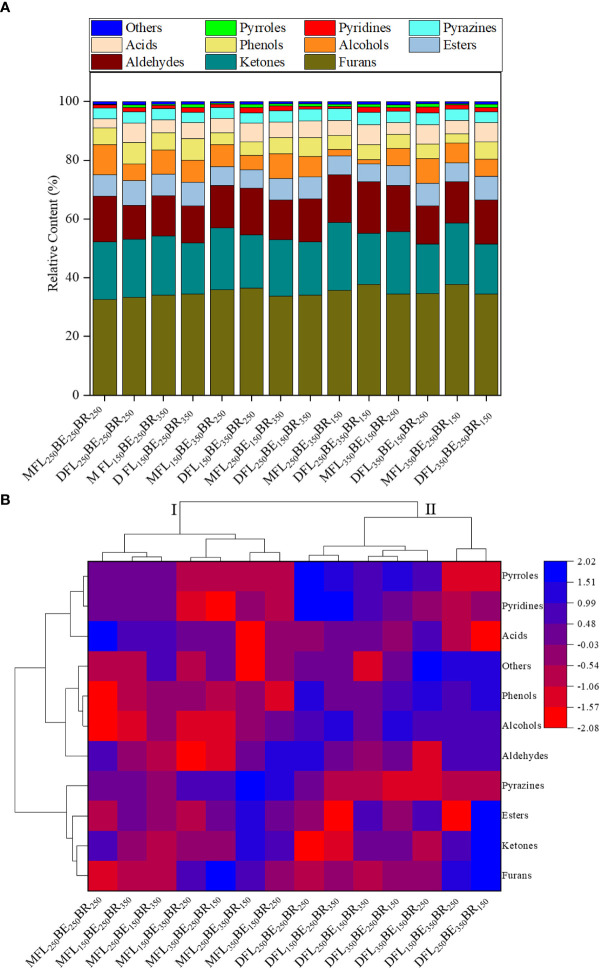
Cumulative graph **(A)** and hierarchical cluster analysis **(B)** of volatile compounds in medium and dark roasted coffee under different fertilization modes. I, the first major cluster; II, the second major cluster. M, medium roasting degree; D, dark roasting degree.

Furans obtained the most abundant relative content (32.71-37.80%) among all volatile compounds in roasted coffee beans, mainly including furfuryl alcohol (increasing with roasting degree), furfural (decreasing with roasting degree) and 5-methylfurfural. The relative contents of furans in MFL_150_BE_350_BR_250_, DFL_150_BE_350_BR_250_, MFL_250_BE_350_BR_150_, DFL_250_BE_350_BR_150_ and MFL_350_BE_250_BR_150_ were all above 35.00% (**Figure 7A** and **Supp. S1**). Pyrazines (16.89-23.08%) are the important flavor compounds whose content was only second to furans, and the relative content of pyrazine compounds under dark roasting showed a downward trend compared with that under medium roasting (**Figure 7A** and **Supp. S1**). Among them, 2,5-dimethylpyrazine, 2,6-dimethylpyrazine, 2,3,5-trimethylpyrazine and 2,3-dimethylpyrazine are important to coffee flavor. Ketones were the third major volatile compounds (11.59-17.66%). Among them, 1-acetyloxy-2-propanone and 1-(2-Hydroxy-5-methylphenyl) ethenone had higher content, while 2,3-butanedione and 2,3-pentanedione that contributed greatly to coffee flavor were in low content (**Figure 7A** and **Supp. S1**). Pyrroles and pyridines accounted for 5.95-8.02% and 3.20-7.42%, respectively, while they had less effect on coffee flavor due to low sensory threshold (**Supp. S1**). Phenolic compounds (2.99-6.83%) increased with the deepening of roasting degree, mainly including maltol, phenol and 4-ethyl-2-methoxyphenol (**Supp. S1**). Acids mainly including acetic acid and isovaleric acid (1.42-10.26%) decreased with the deepening of roasting degree, FL_250_BE_350_BR_150_ obtained low acids content (1.42 -2.05%) due to acetic acid was not detected in this treatment (**Supp. S1**). The acids content in MFL_250_BE_250_BR_250_, MFL_150_BE_250_BR_350_, MFL_250_BE_150_BR_350_ and DFL_350_BE_150_BR_250_ were all more than 8.00% (**Supp. S1**). Furthermore, there was no significant difference on the content of esters (3.51-4.33%) among different treatments (**Figure 7A** and **Supp. S1**).

#### 3.5.2 Cluster analysis of volatile compounds

Cluster analysis was used to visualize the data across a wide range of substances and treatments. As shown in **Figure 7B**, different roasting treatments were clearly divided into two clusters. Cluster I represented medium roasting treatments, which contained high content of acids and pyrazines. Cluster II represented dark roasting treatments, which had a high content of pyrroles, pyridines, phenols, alcohols, aldehydes, and others (**Figure 7B**). This indicated that roasting degree had an important effect on the relative content of volatile compounds. Cluster I contained 2 subcategories. The first subcategory included MFL_250_BE_250_BR_250_, MFL_150_BE_250_BR_350_ and MFL_250_BE_150_BR_350_, which contained high content of pyrroles, pyridines and acids. The second subcategory included MFL_150_BE_350_BR_250_, MFL_250_BE_350_BR_150_, MFL_350_BE_150_BR_250_ and MFL_350_BE_250_BR_150_, which had a high content of pyrazines, esters, ketones and furans. The first subcategory of cluster II was DFL_250_BE_250_BR_250_, DFL_150_BE_250_BR_350_, DFL_250_BE_150_BR_350_, DFL_350_BE_150_BR_250_ and DFL_350_BE_250_BR_150_, in which pyridines and pyrroles were significantly higher than other treatments (**Figure 7B**). Among them, the contents of pyrroles and pyridines were the highest in DFL_250_BE_250_BR_250_ and DFL_150_BE_250_BR_350_, while esters and ketones were low. The second subcategory of cluster II contained DFL_150_BE_250_BR_250_ and DFL_250_BE_350_BR_150_, which had high content of ketones, furans, phenols, alcohols, aldehydes and others (**Figure 7B**).

### 3.6 Cup quality

The sensory indicators of cleanliness, uniformity and sweetness were not displayed in the **Table 2** because they were all 10 points. The scores of aroma, acidity, balance and overall of medium roasted coffee generally ranged from 7.25 to 7.75. Among them, the sensory scores of MFL_250_BE_350_BR_150_ were all higher than 7.40, especially the flavor indicator, which had a great influence on the final total score. However, the sensory scores of MFL_250_BE_150_BR_350_ were low, which ranged from 6.75 to 7.25. In the situation of dark roasted coffee, the body scores ranged from 7.25 to 7.75, with acidity of 6.50 to 7.00. The total scores of medium roasted coffees were in order of MFL_250_BE_350_BR_150_ > MFL_150_BE_350_BR_250_ > MFL_350_BE_250_BR_150_ > MFL_250_BE_250_BR_250_ > MFL_350_BE_150_BR_250_ > MFL_150_BE_250_BR_350_ > MFL_250_BE_150_BR_350_, and the scores were all greater than 80.00 except of MFL_250_BE_150_BR_350_. The total scores of dark roasted coffees were in the order of DFL_350_BE_250_BR_150_ > DFL_250_BE_350_BR_150_ > DFL_350_BE_150_BR_250_ > DFL_250_BE_250_BR_250_ > DFL_150_BE_350_BR_250_ > DFL_250_BE_150_BR_350_ > DFL_150_BE_250_BR_350_, and the total scores of other treatments except DFL_150_BE_250_BR_350_ and DFL_250_BE_150_BR_350_ were above 80.00. Moreover, the total scores of FL_250_BE_250_BR_250_, FL_150_BE_250_BR_350_, FL_150_BE_350_BR_250_ and FL_250_BE_350_BR_150_ under medium roasting degree were higher than dark roasting degree.

**Table 2:** Cup quality of medium and dark roasted beans under different fertilization modes.

Treatment	Aroma	Flavor	Aftertaste	Acidity	Body	Balance	Overall	Total Score
MFL_250_BE_250_BR_250_	7.44ab	7.19bc	7.13b	7.50a	7.13b	7.50a	7.25cd	81.13bc
MFL_150_BE_250_BR_350_	7.31b	7.06cd	7.13b	7.38a	7.13b	7.50a	7.31bcd	80.81c
MFL_150_BE_350_BR_250_	7.56a	7.31ab	7.31ab	7.50a	7.50a	7.50a	7.50ab	82.19a
MFL_250_BE_150_BR_350_	7.44ab	6.88d	6.88c	7.31a	6.81c	7.31a	7.19d	79.81d
MFL_250_BE_350_BR_150_	7.56a	7.44a	7.44a	7.56a	7.44a	7.56a	7.63a	82.63a
MFL_350_BE_150_BR_250_	7.31b	7.25abc	7.13b	7.44a	7.13b	7.50a	7.25cd	81.00bc
MFL_350_BE_250_BR_150_	7.5ab	7.31ab	7.19b	7.56a	7.19b	7.38a	7.44abc	81.56b
Significance test (*P* value)	0.020	<0.001	<0.001	0.266	<0.001	0.340	0.001	<0.001
DFL_250_BE_250_BR_250_	7.13bc	7.13ab	7.56a	6.81b	7.56ab	7.13a	7.19ab	80.50bc
DFL_150_BE_250_BR_350_	7.00c	7.00b	7.19b	6.75b	7.44ab	7.19a	7.13ab	79.69d
DFL_150_BE_350_BR_250_	7.25ab	7.13ab	7.19b	6.69b	7.38b	7.13a	7.25ab	80.00cd
DFL_250_BE_150_BR_350_	7.13bc	7.13ab	7.06b	6.69b	7.56ab	7.25a	7.06b	79.88cd
DFL_250_BE_350_BR_150_	7.44a	7.25ab	7.44a	7.00a	7.44ab	7.13a	7.31a	81.00ab
DFL_350_BE_150_BR_250_	7.13bc	7.13ab	7.44a	6.81b	7.63a	7.13a	7.31a	80.56bc
DFL_350_BE_250_BR_150_	7.38a	7.31a	7.56a	6.81b	7.63a	7.25a	7.31a	81.25a
Significance test (*P* value)	0.003	0.309	<0.001	0.007	0.078	0.451	0.032	<0.001

M, medium roasting degree; D, dark roasting degree. Different lowercase letters in the same column under the same roasting degree indicate a significant difference at *P*<0.05 level.

### 3.7 Correlation analysis of bean nutrients and volatile compounds/cup quality

**Figure 8:**
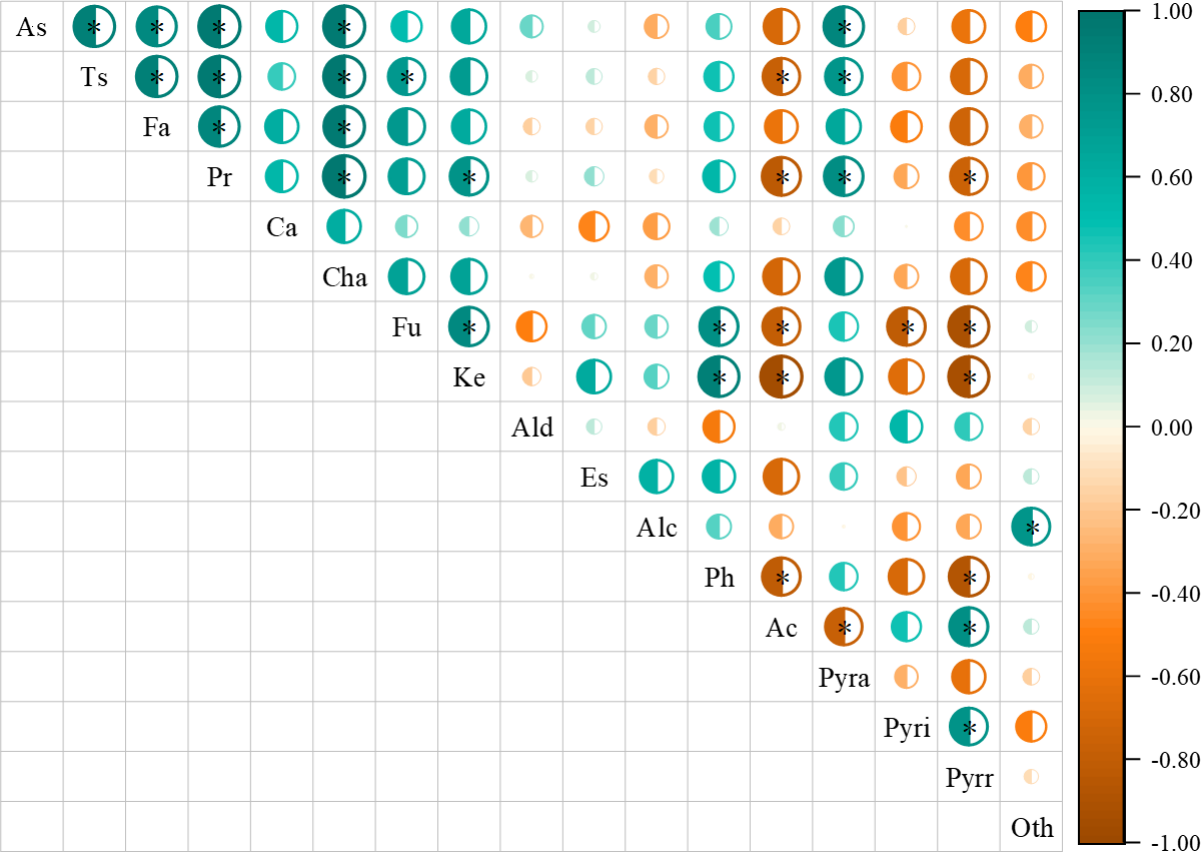
Correlation analysis of nutrients in green coffee beans and volatile compounds in roasted coffee beans. As is ash; Ts is total sugar; Fa is fat; Pr is protein; Ca is caffeine; Cha is chlorogenic acid; Fu is furans; Ke is ketones; Ald is aldehydes; Es is esters; Alc is alcohols; Ph is phenol; Ac is acids; Pyra is pyrazines; Pyri is pyridines; Pyrr is pyrroles; Oth is others. *denotes significant correlation between indicators (*P*<0.05).

Correlation analysis found that nutrients were correlated with flavor compounds to some extent (**Figure 8**). Total sugar content was positively correlated with furan and pyrazine content, while negatively correlated with acid content. Protein content was positively correlated with ketone and pyrazine content, while negatively correlated with acid and pyrrole content. Furthermore, there were interactions between volatile compounds.

**Figure 9:**
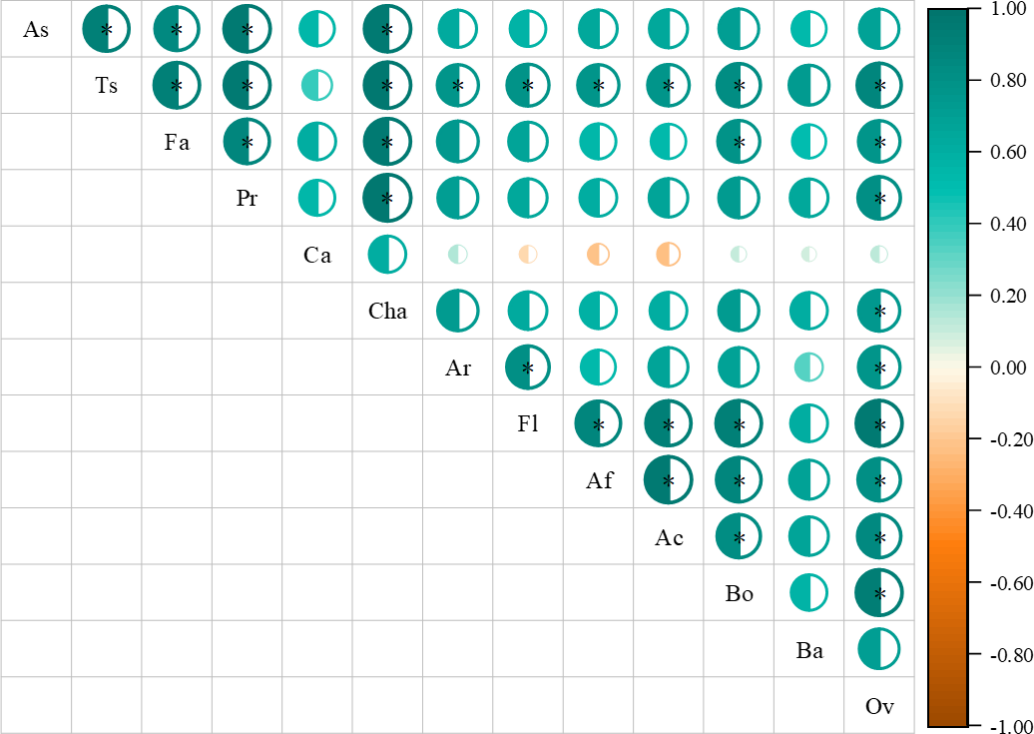
Correlation analysis of nutrients in green coffee beans and cup quality in roasted coffee beans. As is ash; Ts is total sugar; Fa is fat; Pr is protein; Ca is caffeine, Cha is chlorogenic acid. Ar is aroma; Fl is flavor; Af is aftertaste; Ac is acidity; Bo is body; Ba is balance; Ov is overall. *denotes significant correlation between indicators (*P*<0.05).

By exploring the correlation of nutrients content and cup quality, we found that total sugar content was positively correlated with aroma, flavor, aftertaste, acidity, body and overall indicator, fat content was positively correlated with body and overall indicator, chlorogenic acid content was positively correlated with overall indicator, and some sensory indexes influenced each other (**Figure 9**). Overall, there was no direct correlation between nutrients and sensory scores, while there was a clear correlation between green bean nutrients content.

### 3.8 Comprehensive evaluation by TOPSIS

The TOPSIS method was used to comprehensively analyze and evaluate the *A*_net_, *T*_r_, *g*_s_, LWUE, CE, bean yield, bean quality and coffee cup quality indicators of different fertilization modes in 2021-2022. The results are shown in the **Table 3**, and detailed information is shown in **Supp. S2**. As can be seen from the **Table 3**, the ranking order was: FL_250_BE_350_BR_150,_ FL_150_BE_350_BR_250_, FL_350_BE_250_BR_150_, FL_250_BE_250_BR_250_, FL_150_BE_250_BR_350_ and FL_250_BE_150_BR_350_. This indicated that FL_250_BE_350_BR_150_ could provide sufficient nutrients during the fertilizer-demanding period of coffee, which can not only promote the growth of Arabica coffee, but also improve the yield and quality of coffee beans. Therefore, FL_250_BE_350_BR_150_ was the optimal drip fertigation mode.

**Table 3:** TOPSIS comprehensive analysis of different fertilization modes of Arabica coffee.

Treatment	$D_{i}^{+}$	$D_{i}^{-}$	*R_c_*	Ranking
FL_250_BE_250_BR_250_	0.00	0.00	0.48	4
FL_150_BE_250_BR_350_	0.01	0.00	0.40	5
FL_150_BE_350_BR_250_	0.00	0.01	0.68	2
FL_250_BE_150_BR_350_	0.01	0.00	0.16	7
FL_250_BE_350_BR_150_	0.00	0.01	0.79	1
FL_350_BE_150_BR_250_	0.01	0.00	0.28	6
FL_350_BE_250_BR_150_	0.00	0.00	0.53	3

$D_{i}^{+}$ , Positive ideal solution distance; $D_{i}^{-}$ , negative ideal solution distance; R_c_, relative proximity coefficient.

## 4 Discussion

### 4.1 Leaf gas exchange of Arabica coffee

Under the conditions of drip irrigation, rational fertilization modes can timely and effectively meet the demand of crops for soil water and nutrients, improve the flower bud differentiation rate, delay leaf senescence, and prolong leaf gas exchange time, thus improving the photosynthetic capacity of plants (Bartholo et al., 2003; Zhang et al., 2017; Liu et al., 2019). The study found that compared with FL_250_BE_250_BR_250_, increasing fertilizer amount under the same irrigation level during FL and BE period was beneficial for the rapid increase in *A*_net_, *T*_r_, and *g*_s_, while change of fertilization at BR had a little effect on *A*_net_, *T*_r_ and *g*_s_. It might be because the trees possess vigorous life activities at BE and BR, which require massive levels of nutrients. However, the leaf senescence and the decrease of key photosynthetic enzyme activities at BR result in the weakening of leaf gas exchange (Bartholo et al., 2003; Rakocevic et al., 2020; Sun et al., 2022). The BE is vital for the formation of coffee cherry. Increasing fertilizer application under the condition of sufficient soil water is beneficial to the absorption and accumulation of plant nutrients, which increase the leaf area, chlorophyll content and the synthesis of photosynthetic enzymes, thus effectively improving leaf gas exchange at BE and promoting the accumulation of photosynthetic products (Vinecky et al., 2017; Sun et al., 2018). By comparing the average *A*_net_ over two years, it was found that FL_350_BE_250_BR_150_ obtained the maximum value of *A*_net_ and *g*_s_, and FL_150_BE_250_BR_350_ obtained the minimum value of *A*_net_ and *g*_s_. This further indicates that the FL and BE are the key phenophases of coffee photosynthesis, and more nutrients are needed to lay the foundation for high quality and high yield of Arabica coffee.

### 4.2 Dry bean yield and water use efficiency of Arabica coffee

Under drip fertigation, split fertilization at different growth period can promote the transportation and absorption of nutrients in plants, enhance cell metabolism and carbohydrate synthesis, and promote the formation of coffee yield and quality (Vinecky et al., 2017). F_I250_F_II350_F_III150_ obtained the highest dry bean yield, while F_I250_F_II150_F_III350_ obtained the lowest dry bean yield. This indicates that the yield of coffee beans is closely related to the amount of fertilizer applied in different growth periods, especially at FS (Bruno et al., 2011; Sun et al., 2018). Fertilization at phenophases and periods of maturation can timely supplement and balance the nutrient demand of coffee organs, and sufficient water supply can simultaneously promote nutrients release, thus promoting nutrient cycling and absorption in plants, accelerating the synthesis of cytokinin, promoting fruit expansion and weight of single fruit (Parvizi and Sepaskhah, 2015; Liu et al., 2019). In addition, the vigorous physiological activities and strong photosynthetic capacity of coffee trees at BE may be conducive to the accumulation of photosynthetic products (Silva et al., 2003; Zhang et al., 2017). The increase of fertilization at FL can promote the blossom, improve fruit setting rate, accelerate the growth of new shoots, and lay the foundation for the reproductive growth of coffee. We all know that excessive or insufficient fertilization is not conducive to yield production. In this study, FL_250_BE_350_BR_150_ simultaneously obtained the maximum WUE and the highest bean yield, indicating that this kind of fertilization pattern met the growth demands of coffee. Therefore, it is necessary to apply fertilizer by period according to crop fertilizer requirement.

### 4.3 Nutritional components of coffee beans

Soil nutrients can be absorbed and utilized by plants through microbial decomposition and transformation. One-time fertilization is easy to cause the overload operation of soil microorganisms, which may reduce the utilization rate of soil nutrients (Sun et al., 2022). Fertilization on demand according to crop growth period can improve soil microbial metabolism ability and increase soil nutrient availability, thereby improving fruit nutritional quality (Dinesh et al., 2010; Sakai et al., 2015). In addition, drip fertigation can provide accurate application of water and fertilizer, reduce nutrient leaching, well maintain the loose state and porosity of soil, thus improving soil physical and chemical properties (Sakai et al., 2015; Yan et al., 2021). In this study, FL_150_BE_350_BR_250_ and FL_250_BE_350_BR_150_ significantly increased the content of ash, total sugar, fat, protein, caffeine and chlorogenic acid compared with FL_250_BE_250_BR_250_. However, FL_250_BE_150_BR_350_ and FL_350_BE_150_BR_250_ significantly decreased the content of ash, total sugar, fat, protein and chlorogenic acid compared with FL_250_BE_250_BR_250_. On the one hand, split fertilization according to growth period may affect the decomposition and conversion rate of nutrients by soil microorganisms, thus affecting the availability of nutrients (Sakai et al., 2015; Vinecky et al., 2017). On the other hand, the BE is the critical period for the formation of nutritional quality of coffee cherry. The amount of fertilizer applied during this period directly affects the nutrient transport and distribution in coffee trees, and the formation and accumulation of photosynthetic products (Zhang et al., 2017; Sun et al., 2018; Wu et al., 2019). It was found that nitrogen fertilizer was closely related to the synthesis of amino acids in fruit. Appropriate amount of nitrogen fertilizer could increase the contents of caffeine, protein and chlorogenic acid and the yield of coffee beans (Wang et al., 2008; Liu et al., 2016). Phosphorus is involved in plant photosynthesis and nitrogen absorption and metabolism, and adjusts the proportion of nutrients in plants, thus regulating the content of chlorogenic acid in fruits (Wang et al., 2008). Potassium fertilizer can enhance enzyme activity in plants, participate in regulating CO_2_ fixation in photosynthesis, and promote sugar metabolism and protein synthesis, thus improving fruit quality (Ali et al., 2021). Therefore, fertilization according to the nutrient requirements of Arabica coffee can coordinate the growth of vegetative organs and reproductive organs, and the accumulation and distribution of assimilates, so as to achieve the purpose of improving the nutrient content of coffee beans.

### 4.4 Volatile compounds of roasted beans

In addition to the stimulation effect and the health care function of caffeine, the popularity of coffee is mainly attributed to the unique aroma and flavor of volatile compounds in roasted coffee (Dulsat-Serra et al., 2016). Furans with sweet, caramel and barbecue tastes are associated with the thermal decomposition of sugars, the Maillard reaction of reducing sugars and amino acids, and the oxidation of polyunsaturated fatty acids (Rocha et al., 2004; Sunarharum et al., 2014; Zakidou et al., 2021). In this study, the content of different furans varied with roasting degree, which might be related to the decomposition conditions of precursors and the further decomposition of furans with the deepening of roasting degree. Petisca et al. (2014) found that furfural and its derivatives would further decompose into furoic acid under heating conditions, and then decarboxylated to produce CO_2_. Cluster analysis showed that FL_150_BE_350_BR_250_ and FL_250_BE_350_BR_150_ had high relative content of furans under different roasting degrees, which might be related to the high total sugar and protein content in these two treatments. Correlation analysis results also indicated that total sugar content was positively correlated with furan content. The formation of pyrazines is related to the Maillard reaction and the pyrolysis of amino acids (Sunarharum et al., 2014). Pyrazines show attractive coffee or cocoa fragrance, and its third-generation derivatives have barbecue and earthy tastes (Toci and Boldrin, 2018). The content of pyrazines in the baking process depends on the ratio of amino acids to sugars in the reaction system (Toci et al., 2020). In this study, pyrazine compounds had high relative content under medium roasting degrees (M), which might be due to the fracture of the six-membered ring of pyrazines under dark roasting degrees (D), resulting in the formation of olefins and then volatilization (Kiefer et al., 1997). The relative contents of pyrazines in MFL_150_BE_350_BR_250_, MFL_250_BE_350_BR_150_, MFL_350_BE_150_BR_250_ and MFL_350_BE_250_BR_150_ were higher than that under dark roasting degrees, which might be due to the high protein content in these treatments. Moreover, the high pyrazine content in FL_350_BE_150_BR_250_ might be attributed to the defective coffee beans, which resulted in the content of free amino acids far exceeding that of carbohydrates, thus more pyrazines were generated through the Maillard reaction (Toci et al., 2020). Correlation analysis found that pyrazine content was positively correlated with total sugar and protein content, which further explained the relationship among the three substances. The formation of ketones is related to Maillard reaction or Strecker degradation (Sunarharum et al., 2014; Toci et al., 2020). The high content of ketones in FL_250_BE_350_BR_150_ might be related to the high protein content. Acids affect the flavor and taste of coffee, and tend to decompose at high temperature. The high content of acids in MFL_250_BE_250_BR_250_, MFL_150_BE_250_BR_350_ and MFL_250_BE_150_BR_350_ might be closely related to the nutrient content and defective beans in coffee (Toci et al., 2020). Many studies have shown that the precursors of green coffee beans are closely related to the flavor compounds of roasted coffee (Sunarharum et al., 2014; Hu et al., 2020; Zakidou et al., 2021). This study found that there were differences in coffee volatile compounds among different treatments. The main reason may be that fertilization amount at different periods affected the tree growth and physiological activity, which changed the nutritional components of beans.

### 4.5 Cup quality of roasted beans

Cup quality directly affects the price of coffee. A high-quality cup is a balanced combination of flavor, body and aroma (Sunarharum et al., 2014). Due to the cultivar, growing environment, processing method and roasting degree, the composition and content of coffee are different, which directly affects the taste, aroma, color and quality of roasted coffee. In this study, the high scores of coffee aroma, acidity and balance of medium roasted beans might be related to the ketones and acids generated by proteins and sugars under medium roasting degrees. The correlation analysis indicated that protein content was positively correlated with ketone content, and sugar content was positively correlated with coffee aroma and acidity score. In addition to being a sweet substance, sugar is also conducive to the formation of coffee aroma and flavor (Sunarharum et al., 2014; Zakidou et al., 2021). It has been found that coffee cups with high sugar content also have high quality (Barbosa et al., 2019). The body score of coffee under dark roasting degrees was higher than that under medium roasting degrees, which might be related to the increasing fat content under dark roasting degrees. Vasconcelos et al. (2007) found that the maximum fat content of Arabica during the baking process was 11.18% higher than that of green coffee beans. Correlation analysis showed that fat content was positive correlation with body score. The release of volatile compounds such as aldehydes, ketones and alcohols from fat during baking process affects the body of coffee, thereby influencing the cup quality (Barbosa et al., 2019). Only the quality score of FL_250_BE_150_BR_350_ was below 80.00 under different roasting degrees. This might be due to the low total sugar and fat content of some green beans, which led to the fact that various volatile substances of roasted coffee beans did not reach the equilibrium point, thus affecting the cup quality.

## 5 Conclusions

Increasing fertilization ratio at FL and BE obviously improved the average *A*_net_, *T*_r_, *g*_s_ and CE. FL_250_BE_350_BR_150_ significantly increased bean yield, WUE and PFP, while FL_250_BE_150_BR_350_ and FL_350_BE_150_BR_250_ significantly decreased those indexes. FL_150_BE_350_BR_250_ and FL_250_BE_350_BR_150_ increased contents of total sugar, fat, protein, caffeine and chlorogenic acid compared with other treatments. Medium roasted coffee beans contained high levels of acids and pyrazines, while dark roasted coffee beans contained high levels of pyrroles, pyridines, phenols, alcohols, aldehydes and other compounds. MFL_250_BE_350_BR_150_ and DFL_350_BE_250_BR_150_ obtained the highest total cup score. Correlation analysis showed that bean nutrient contents were closely related to volatile and cup quality. The TOPSIS indicated that FL_250_BE_350_BR_150_ was the best fertilization mode. The experiment can provide some practical reference for coffee cultivation. Further study may focus on the relationship between soil environment and coffee quality and volatile components.

## Data availability statement

The original contributions presented in the study are included in the article/**Supplementary Material**. Further inquiries can be directed to the corresponding authors.

## Author contributions

RL: Investigation, Formal Analysis, Visualization, Writing Original Draft. JC: Investigation, Formal Analysis, Visualization. XL: Conceptualization, Methodology, Writing Review and Editing, supervision. ZW: Writing Review and Editing, Visualization. HL: Resources, Writing Review and Editing. JG: Methodology, Writing Review and Editing. HW: Methodology, Writing Review and Editing. NC: Conceptualization, Methodology, Writing Review and Editing, supervision. LZ: Writing Review and Editing, supervision. All authors contributed to the article and approved the submitted version.
